# Nuancing ‘Emotional’ Social Play: Does Play Behaviour Always Underlie a Positive Emotional State?

**DOI:** 10.3390/ani14192769

**Published:** 2024-09-25

**Authors:** Giada Cordoni, Ivan Norscia

**Affiliations:** Department of Life Sciences and Systems Biology, University of Torino, 10123 Turin, Italy

**Keywords:** cooperation, competition, positive/negative emotional states, aggressive play, play signals, rapid motor mimicry

## Abstract

**Simple Summary:**

We review the existing research on social play in humans and other mammals to highlight the complex nature of this behaviour, which is regulated by various hormones and neural circuits. Play can swing from cooperation to competition. Contrary to what has been previously thought, when animals (including humans) play, they might not always be in a positive emotional state or in a relaxed context. By checking examples from human and non-human mammals, we aim to examine current tools and methodological approaches that can give information about the different individual emotional states possibly driving playful interactions and about the individual, socio-ecological, and structural factors potentially revealing the emotional nature of the play. We discuss the context in which play occurs (relaxed/not relaxed) and the structural similarities between play fighting and aggressive behaviour, considering how more competitive forms of play can serve as alternatives to aggression. Additionally, we look at the postures/movements and facial expressions (e.g., play faces) used as signals to indicate the player’s intent and at copying behaviours, like rapid motor mimicry, which can enhance synchronization, non-agonistic competition, and possibly emotional sharing between players.

**Abstract:**

This review focuses on social play, a complex behaviour that is often difficult to categorize. Although play has been typically associated with positive emotional states, a thorough examination of the literature indicates that it may relate to different emotional systems, from attachment to conflict. Play oscillates between competition and cooperation, and includes a spectrum in between; thus, quantitatively identifying and demonstrating the emotional nature of play remains challenging. We considered examples from human and non-human animal studies and explored the emotional and neuro-hormonal systems involved in play. We assessed ethological data possibly indicating the emotional states underlying play, and we focused on the cooperative and competitive elements of play. We investigated the relationship between play and affiliative/aggressive behaviours, the communicative meaning of play signals (especially primate play faces), and the motor and possibly emotional contagion function of rapid motor mimicry during play. From all the literature on play, this review selects and combines studies in an innovative way to present the methods (e.g., play indices and social network analysis), tools (e.g., sequential analysis and facial coding software), and evidence indicative of the emotional states underlying play, which is much more complex than previously thought.

## 1. Introduction

‘*The essence of play is paradox*’ [1] (p. 471)

This review focuses on what is probably one of the most complex and puzzling behaviours: social play.

Understanding when two animals are playing is not straightforward, and the literature does not always explicitly state the criteria used to determine if certain interactions are playful or not. It has been often pointed out that play is defined by what it is not, rather than by what it is, because it borrows patterns from other behavioural contexts, such as aggression and affiliation [2,3]. Criteria have been established to define when an interaction is playful [4] and when it involves play fighting [5]. Play is a voluntary and rewarding activity with no apparent adaptive function, characterized by repeated (but not stereotyped), incomplete, and exaggerated behavioural patterns [4]. In particular, during play fighting individuals do not protect specific resources and they adopt behavioural strategies (i.e., role reversal and self-handicapping) and avoid causing injuries to their partners to maintain play. Players can also perform specific vocalizations, postures, and expressions to communicate their non-aggressive intent [5]. In this review, we investigate play based on these parameters.

In the literature, studies investigating which emotional states might drive play are still limited. Although play has typically been associated with positive emotional states [6], emerging knowledge indicates that play can be associated with various emotional systems, ranging from attachment to conflict. Indeed, play oscillates between competition and cooperation, between attack/flight situations and calm [7]. Although during play—especially competitive play—specific play signals such as the play face [8,9,10,11] are used, much remains to be done to understand how these can provide information about the emotional valence of play. Hence, demonstrating these aspects quantitatively from a behavioural perspective is still a challenge.

In this review, we aim, for the first time, to gather evidence on the multifaceted emotional nature of play and, more importantly, to identify which elements of play can inform its nature, how these elements can be detected, and through which tools.

The objective of this review is to provide methodological suggestions to investigate the characteristics of social play in a quantitative and reproducible manner. We focus on both macro (e.g., frequency, distribution, and level of competitiveness) and micro (e.g., play face duration and replication) characteristics of play, to understand the emotional states underlying it. To reach this goal, we first explore the emotional and neuro-hormonal systems that generate, guide, and modulate play behaviour, through the complex, albeit still partial, framework provided by the literature. We then analyse ethological data possibly informing about the emotional state that ‘animates’ play, focusing, due to the limited number of studies in this respect, on the cooperative and competitive nature of play. This may indicate, respectively, positive emotional states (associated with affiliative elements of play) or negative emotional states (associated with the aggressive elements of play). In this regard, we seek to understand in which way the ‘play’ system can be associated with affiliative and aggressive behavioural systems, and, more importantly, how this association can be investigated. We then delve into the play sessions, particularly focusing on their structure and play signals. In the last decade, the development of low-cost but high-quality audio–video recording devices has enabled the investigation of play interactions using frame-by-frame or slow-motion analysis, allowing for the examination of playful behavioural patterns and their exchange within sessions. From a methodological perspective, new data analysis approaches (e.g., structural index calculation such as variability or repetition indices) and new tools (e.g., sequential analysis or facial coding software) allow quantitative measurement of the structure of play, the level of responses to play signals (e.g., rapid facial mimicry), and the sequence of actions before or after these signals. These methods/tools may permit inference on the positive or negative emotional states of individuals engaging in play.

We acknowledge that the spectrum from negative to positive states is complex and includes a graduated range of emotional states, but in this review we use what is currently known and published.

## 2. Social Play: What Are the Underlying Emotional Systems?

**Figure 1:**
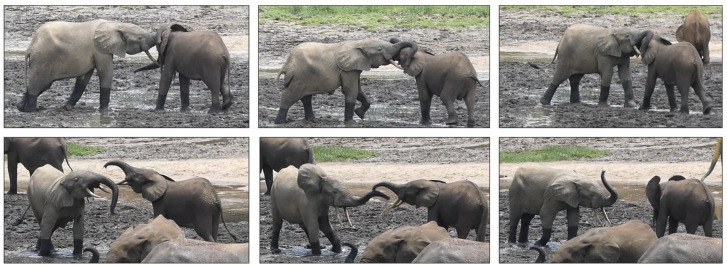
Sequence of play patterns during a playful interaction between immature African forest elephants (*Loxodonta cyclotis*) in the National Park of Dzanga-Sangha (Central African Republic). Screenshot: Giada Cordoni. Edited by Giada Cordoni.

Play behaviour may have independently evolved and transformed across different taxa [3]. While play may not have a direct function in individual survival or fitness success, it can still provide immediate or delayed benefits to the individuals involved. To address this apparent inconsistency, it is useful to examine the conditions under which play behaviour first evolved in ancient animals and their modern descendants as well as the secondary processes that, in an earlier evolutionary period, provided physiological, behavioural, social, cognitive, or emotional advantages to individuals engaging in play [4]. Based on Burghardt’s model [4], in some lineages play-like behaviour could emerge from the incomplete development of other functional behavioural systems (primary process play). Subsequently, in some of these lineages a play behaviour system evolved through the reorganisation of patterns typical of other systems, such as anti-predatory, aggressive or feeding behaviours (secondary process play). Finally, certain lineages have integrated patterns from different systems into a super-play system (tertiary process play). Therefore, play, especially in its social form, results in highly flexible behaviour in terms of function, structure, and communicative signals (Figure 1).

**Figure 2:**
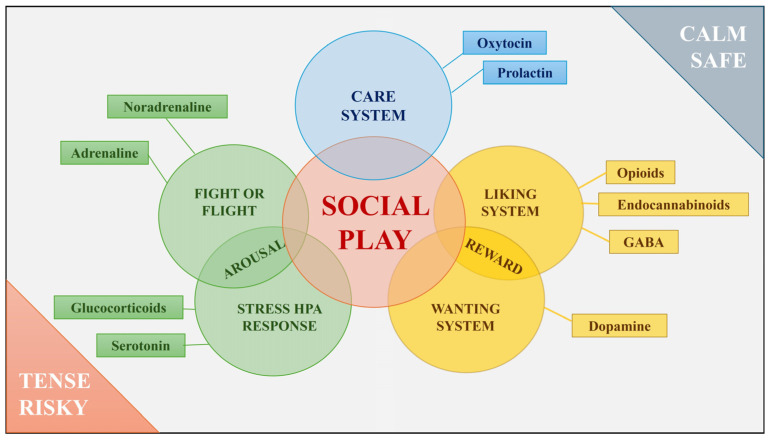
Graph summarizing the relationships between social play and neural pathways, hormones, and neurotransmitters associated with it. Graph by Ivan Norscia.

This flexibility is in line with the Polyvagal Theory, according to which a phylogenetic shift in neural regulation of the autonomic nervous system passes through different stages, each with an associated behavioural strategy, including social play [7,12]. Social play would result from a neural exercise that requires the ability to swing between a fight-or-flight response, related to the arousal emotional systems, under tense or risky situations, and social cooperation, related to care and reward emotional systems, under calm, safe conditions [7,12]. Despite social play being classically associated with an emotion of joy [6], the emotional states that lead to the onset of social play are more multifaceted and articulated. Consistently, different studies in rats have found that social play is modulated by neurohormones involved in different emotional macrosystems. In particular, opioid, endocannabinoid, GABA, and dopamine are associated with the reward system of motivation and pleasure (among others); oxytocin and prolactin are linked to the care system; and testosterone, noradrenaline, serotonin, and glucocorticoids are broadly related to the arousal systems, from anger/rage (fight or flight) to HPA (the hypothalamic-pituitary–adrenocortical axis) regulation in the stress response [13,14,15,16,17,18]. These systems are intertwined (e.g., dopamine is involved both in the stress response and connected with the oxytocinergic circuit [14,17]), and play’s flexible nature is regulated by the coordinated activity of a corticolimbic structure network [15,19,20,21,22]. Hence, depending on the context, social play may underlie different emotional systems not necessarily associated with a positive affect (see Figure 2). Below, after briefly considering the neurobiological foundation of the emotional systems underlying play, we consider the behavioural aspects that support the association between play and such social systems.

### 2.1. Play and Reward

The neural systems involved in the rewarding properties of food, sex, and drug abuse also modulate the expression of social play [17,23]. Reward processes can be dissociated into different components: pleasurable properties, incentive motivational properties, and effects on learning [17,23]. These components are mediated by different neural systems, such as the prefrontal cortex, striatum, and amygdala, and neurotransmitter systems (e.g., dopamine, opioids, cannabinoids, and GABA) [17,23]. For example, the nucleus accumbens, a significant locus for dopamine and opioids, likely plays a pivotal role in modulating play motivation and pleasure (for an extensive review see [15]). In rats, treatment with morphine both increases the initiation of play and prolongs playful sessions even though it does not concomitantly enhance feelings of safety or reduce anxiety, thus suggesting that opioids might not fine-tune negative emotional states linked to play [23]. The rewarding property of social play has been demonstrated by operant and place conditioning experiments (particularly in rats) that highlighted the pivotal role of the rewarding aspect of play in stimulating social interactions [15,24,25]. Evaluating whether play is rewarding based on behavioural observations is challenging and requires an advancement in ethological studies. Now, we only have indirect indications that play is rewarding, such as repeated play sessions by the same dyads, the use of play invitation, and prolongation of play sessions by using play signals [26,27,28].

### 2.2. Play and Social Attachment

Oxytocin, the neurohormone that modulates maternal care and social attachment (starting with the mother–infant relation [29,30]), and prolactin [13] can also influence play behaviour. For example, in rats, oxytocin can favour social play in novel contexts (especially in females [31]), and increased oxytocinergic neurons can be associated with reduced play fighting [32]. In juvenile Japanese macaques (*Macaca fuscata*), the play network can be positively correlated with the association network in daily interactions, thus suggesting the pivotal role of playful interactions in strengthening social bonds [33]. In infant spotted hyenas (*Crocuta crocuta*), the increase in play interactions between siblings can promote the integration of pups within the group [34]. In African savanna elephants (*Loxodonta africana*), social play can particularly occur more often between individuals of different families as a means to establish a social bridge and long-term relationships [35,36,37]. It has been proposed that play may serve as a tool, also in adult mammals, to assess and manipulate social relationships [25,26,38], thus favouring the maintenance of brain plasticity throughout the individual’s life [22]. Hence, play may increase levels of familiarity between less bonded individuals (even strangers, e.g., [39]), foster new social relationships, and reinforce existing close social bonds [40,41]). However, establishing new relationships and reinforcing them are two different functions, and, overall, the literature is opaque with respect to the distinction between them. The translocation of an entire group of bonobos (*Pan paniscus*) to another zoo, and its union with the group already resident in the new site, allowed the exploration of this issue [42]. Indeed, it was possible to clearly distinguish between new and already existing social relationships and check how play was used before and after the groups’ merging compared to other bonding behaviours [42]. The study found that, while adult bonobos used socio-sexual contacts, immature bonobos used play to establish new relationships (as their levels were highest between relocated and resident group member dyads). Furthermore, grooming was used to maintain pre-existing relationships (as grooming increased within the individuals of the relocated group). Whether play favours the formation of new bonds or the reinforcement of new ones may change how (and for what) play is used, and future investigation may focus on this understudied aspect.

### 2.3. Play and Arousal

Arousal can quickly lead to the fight-or-flight response and, later, to the activation of the HPA stress axis and related regulatory circuits (e.g., serotonin; [16,17]). With respect to the fight-or-flight response, noradrenergic neurotransmitters, relying on the basolateral amygdala, habenula, and prefrontal cortex and involved in such response, may regulate the structure of play (e.g., session duration; [43,44,45]). A decrease in noradrenaline levels, in parallel with cortical and subcortical processes, may reduce social play. Furthermore, noradrenergic processes may affect some cognitive mechanisms by which social play experience can influence future behaviours (see an extensive review [22]). Play fighting, aggression, and dominance in male mammals can also be related to high concentrations of androgens [46]. For example, early exposure to testosterone may provoke an increase in play fighting frequencies in both rodents [47] and primates (humans [48]; non-human primates [49,50]). In juvenile rats, lesions on the lateral septum—housing receptors for gonadal hormones (among others [21])—can provoke the enhancement of competitive play fighting in both sexes [51]. Interestingly, in adult rats the same lesion increased aggressive behaviours that morphologically resemble play fighting seen in juvenile individuals, albeit with notable differences such as the absence of behavioural inhibition present in play [51].

With respect to other types of arousal-related responses, the serotonergic circuit interacts with the HPA stress axis activation and can therefore regulate stress responses [52]. In rats, pre-natal exposure to a serotonin reuptake inhibitor can prevent the negative effects of maternal stress on play frequency between siblings. On the other hand, this reuptake inhibitor can increase ‘aggressive’ play levels between unfamiliar conspecifics [53]. Furthermore, some evidence suggests that serotonin may fine-tune the influence of dominant–subordinate relationships during social play [22].

An increase in anxiety levels may be related to increased secretion of cortisol causing changes in behaviours, including play [54,55,56,57]. For example, in marmosets (*Callithrix geoffroyi*), individuals exposed to elevated cortisol concentrations during the pre-natal period then showed a decreased propensity to engage in play [55]. On the other hand, social play can affect stress levels. For example, social play deprivation in juvenile hamsters can negatively affect neuronal development in the ventromedial prefrontal cortex and increase vulnerability to social stress effects in adulthood [58]. From a behavioural standpoint, the connection between play and stress has been found in different cases, from rodents to primates. In rats, Klein and co-authors [59] demonstrated that under acute stress during which individuals experience negative emotional states, social play was suppressed, while it remained unaffected under mild or chronic stress. In adult horses, more playful individuals suffered more from chronic stress than less playful ones; indeed, play frequencies positively correlated with levels of chronic stress [60]. Maternal separation—a negative-valence experience—leading to undernourishment or a decline in maternal care has been associated with an increase in play behaviour in kittens, rat pups, and rhesus monkeys (for an extensive review, see [61]). In primates, frequencies of play (in particular, play fighting) can peak in the period of social tension that precedes food distribution characterized by high individual arousal levels (i.e., pre-feeding; chimpanzees, *Pan troglodytes* [62,63]; bonobos, *Pan paniscus* [64]; and lowland gorillas, *Gorilla gorilla gorilla* [65]). In both chimpanzees and bonobos, during pre-feeding the increase of play can mainly involve unrelated dyads, to possibly manage competition risks and promote an individual positive emotional state [62,64].

The above findings, taken altogether, indicate that the inception of social play may not necessarily match with a positive emotional state of the players. On the contrary, under specific conditions, social play may be promoted by an adverse psychological or emotional state and may increase individual emotional resilience [60,65] and the ability to cope with stressful or agonistic contests [60,62,64,66,67]. The above framework points toward a distinction that has been understudied so far, that is, whether play starts under relaxed conditions, or whether it is initiated when individuals are tense, anxious, or mildly stressed and as a tool to restore homeostasis. Play features and dynamics may vary depending on this aspect.

## 3. The Dual Nature of Social Play: Doppelgänger of Aggression?

The first section of this review highlighted that social play could take on different emotional valences, shifting from negative situations linked to fight-or-flight responses or stress to positive ones associated with social attachment. Neurobiologically, social play may arise from non-aggressive arousal, aggressive arousal suppression, and/or as a precursor of aggressive behaviour [21,22,23,24,25,26,27,28,29,30,31,32,33,34,35,36,37,38,39,40,41,42,43,44,45,46,47,48,49,50,51,68]. Owing to its ‘emotional flexibility’, social play is the only behaviour that can variably swing from competition to cooperation, which is particularly adaptive in socially interacting animals, as group living involves a delicate balance between cooperation and competition. The tension between the interests of the group and the individual has possibly favoured evolutionary transitions that have shaped social play use and features depending on whether it can replace aggressive competition to manage the conflict of interest over resources or to affiliate with others to cooperate [69].

### Play Fighting or Play for Fighting: Aggressive Play or Playful Aggression?

In the psychological and pedagogical literature, the play of pre-school children is often referred to ‘aggressive play’, where playmates enjoyably and voluntarily engage in interactions including aggressive-like actions yet lacking intent to harm either emotionally or physically [70]. Indeed, Boulton [71] found that adults often mistakenly interpreted children’s aggressive interactions as play fighting interactions, and vice versa. They relied on children’s facial expressions and motor action features to correctly discern the nature of the interaction. In humans, during adolescence, compared to the other developmental phases, the border between real and play fighting becomes more blurred [72]. Indeed, restraint and role reversal (i.e., balance in assuming winner–loser role/position during play) are less obvious and frequent; play can be used as a tool to demonstrate either strength or dominance over the companion [5,72]. The same terminology ‘aggressive play’ has also been used for non-human primates, especially in relation to play fighting [73]. In juvenile human and non-human primates, playful teasing (e.g., offer–withdrawal of an object, disrupting others’ activity) is highly ambiguous and competitive. To ensure it is interpreted as non-aggressive behaviour, teasers have to effectively communicate their friendly intent and correctly interpret the behavioural responses of the recipients (for an extensive review, see [74]). ‘Aggressive play’ may favour a self- and social-assessment process [75] through practising and developing physical ability and restraint, acquiring social competence, improving cognitive skills, and training for the unexpected [66,70].

In non-human animals, social play (particularly play fighting) may revolve around competition over diverse targets. For instance, pottos and giant mouse lemurs engage in competitive play to initiate grooming, whereas marmots use play fighting to establish mouth-to-mouth contact, a typical greeting behaviour [41]. Furthermore, in many species play fighting may function as a substitute for real fighting, even if in a (almost) safe context [25,26,41,75]. In pre-weaning domestic pigs (*Sus scrofa*), play fighting rapidly transitioned into real fighting along a continuum, with play fighting frequencies being positively correlated with aggression rather than affinitive frequencies [25]. Moreover, the winner–loser socio-matrices of play positively correlated with the socio-matrices of aggression, thus indicating that in pigs the winners of play fights were also most likely winners of real fights [25]. In lowland gorillas, play fighting showed a peak of frequency among juvenile and adolescent males [65]. By this means, gorilla males gather immediate feedback about their partners’ physical skills, thus testing the fighting abilities of potential future competitors in a ‘non-serious’ context. Compared to bonobos, immature chimpanzees showed a more competitive form of play [76]. Indeed, chimpanzee play fighting escalated more frequently into real fighting, had less duration, and usually did not involve more than two partners concomitantly. In adult chimpanzees, social play and reciprocal grooming were negatively correlated, thus suggesting that play did not necessarily indicate the quality of social relationships between individuals [63]. Cordoni and colleagues [23] demonstrated that adult chimpanzees possessing dominant positions in real fighting maintained such positions in play fighting too. The authors hypothesized that in adult chimpanzees, dyadic dominance relationships can be translated from real into play fighting. To sum up, all these findings may indicate that play may not be that playful after all. Play fighting may replace real fighting under certain circumstances and according to specific individual (e.g., species, sex, and age) and social (e.g., dominance relationships) features.

The distinction between the cooperative and competitive nature of play is not clear-cut as it may depend on context, socio-ecological factors, and individual features (e.g., sex, age, and rank). For example, in African savanna elephants the social network of play is linked to the social network of affiliation only when immature individuals are included but not when only adults are considered [37]. In domestic dogs (*Canis lupus familiaris*), although play levels did not show a sex bias, the self-handicapping strategy was less present during male–male playful interactions. This suggests a possible use of play as a safe way for intrasexual competition [77]. Among humans, in the African Bofi forager population, subsisting on cooperative hunting/gathering activity, children performed a more cooperative type of physical and object play compared to children belonging to the Bofi farmer population, subsisting on individual horticulture/trading activity [78].

In sum, part of the literature highlights the competitive nature of play in various situations. Play, particularly in adults, has been associated with the evolution of tolerance in relation to its cooperative value, in conditions of reduced competition—for example, in Verreaux’s sifaka (*Propithecus verreauxi*) lemurs compared with the more despotic ring-tailed lemurs (*Lemur catta*) [39], or in bonobos compared to the less tolerant chimpanzee [63,76]. We propose that it is the competitive use of play, among adults or subadults, resulting from certain neurophysiological processes (e.g., reduced testosterone [37]) that can especially promote tolerance, resulting in a reduction in levels of overt aggression. In this respect, the conflict of interest that necessarily arises in social groups [69] is managed through competitive play rather than aggression, which results in social tolerance. This can trigger a positive feedback loop that replicates tolerant behaviours towards others. These aspects, in our opinion, need to be better analysed in future studies as it is also important to distinguish the characteristics of play among adults from those of play that simply involves adults (with juveniles), because the emotional value of play may vary.

## 4. Play and Its Structure

From a structural standpoint, play results in a puzzling behaviour since it recruits and recombines motor patterns from other behavioural systems [3,4]. In many mammals, the motor patterns performed during play fighting largely reflect those used in real fighting. Play fighting may provide practice of tactics that are similar to those used in real fighting, although it does not completely mirror real fighting, especially in the way motor patterns are performed [4,73,79]. Two out of Burghardt’s five criteria [4] state that during play—unlike in ‘serious’ behaviours—animals perform exaggerated and repeated motor patterns. Nevertheless, the manner in which a playful interaction is performed can lead to a more cooperative or competitive form of play and, therefore, inform on the underlying emotional valence of play. Specific indices have been developed to quantitatively evaluate the degree of cooperativeness/competitiveness of a playful interaction and, recently, the measurable distinction between play and other, ‘serious’ behaviour, such as aggression.

The Play Asymmetry Index was first used by Cordoni and colleagues [80] to quantify the extent to which play is ‘imbalanced’, and therefore competitive, between players. It depends on the numbers of offensive (e.g., play push, play pull, play slap) and defensive (e.g., play shelter, play wriggle, play flee) playful patterns exchanged between players [25,26]. The asymmetry occurs when one individual actively achieves or maintains a dominant/offensive position over their playmate for most of the session [77]. The degree of play asymmetry can vary according to individual (e.g., species, sex, and age) and social factors (e.g., dominance relationship and quality of social bond [81]). For example, in domestic dogs, play asymmetry can increase as puppies grow older [77,82,83]. In juvenile coyotes living in the same litter, play asymmetry increases during interactions between dominant and subordinate individuals [82]. On the other side of the coin, in some species the asymmetry level is higher in real rather than in play fighting thus indicating that real fighting maintains the highest degree of directionality (domestic pigs ([25,84]; chimpanzees [26]). Indeed, asymmetrical aggressive events are crucial to acquiring a dominant status as the ranking position of an individual within a group increases as the number of agonistic encounters consistently won by this individual increases [85,86].

Repetitiveness and pattern variability may be other features that allow the distinction between playful and ‘serious fight’. These features can be quantitatively measured by the Repetition (RI) and Repeatability of Same Behaviour (RSBI) indices and the Shannon Index (H’), respectively [25,26,84]. RI and RSBI are calculated to evaluate the level of repetition of the same motor pattern within a single play session. H’ is the most common index used in ecological studies to evaluate the level of biological diversity [87,88], but it has been adapted for the first time in the context of play behaviour by Cordoni and colleagues [26] to measure the level of play variability in terms of different types of motor patterns performed within a single session [25,26,84]. Since then, the new use of the index has been adopted by other studies, such as Maglieri and Palagi [89], although the authors refer to its original use within the mathematical theory of communication [90] rather than the modified use to determine play diversity [84]. Degrees of repetitiveness and variability are generally higher in play than in aggressive interactions. Therefore, they can be useful tools to measure the level of competitiveness of play. For example, in piglets, play fighting sessions were less variable than in immature wild boar hybrids, whereas asymmetry was comparable [84]. Hence, piglets used play in a less cooperative way compared to their wild counterpart (wild boar), which may be related to the strongly competitive nature of play fighting in piglets, which can be used as a substitute for aggression.

In general, evaluation of the structure of playful interactions compared across more and less docile species (e.g., the wild and the domestic counterparts) and/or with homologous aggressive interactions (e.g., play vs. real fight) can provide a valid tool to assess whether play is really ‘playful’. Studies on play structure are still in their infancy, but future investigation should delve deeper into this topic to assess the affective states that underpin play.

## 5. Playful Signals: What Kind of Message Do They Convey?

**Figure 3:**
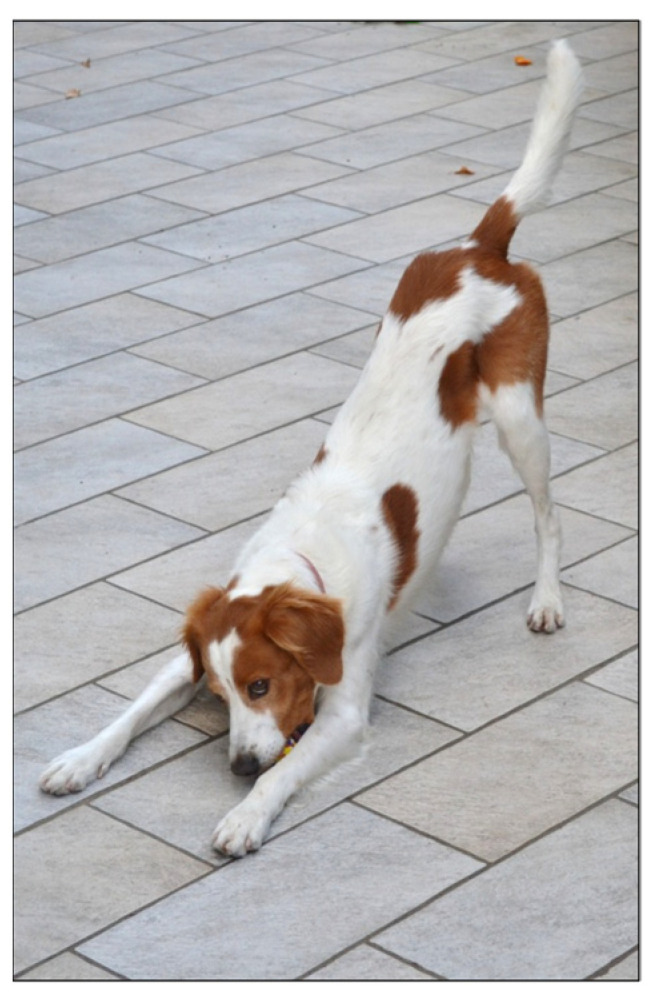
An example of a play bow, a typical play signal in canids. The dog (*Canis lupus familiaris*) crouches on its forelimbs, remains standing on its hind legs, wags its tail, and sometimes barks [80,94,95]. Personal photo by Giada Cordoni.

Specific structural features of play are not the sole distinguishing traits that can be used to understand its competitive or cooperative essence and the possible emotional drive. During playful interactions, animals employ body posture, movements, facial expressions, and vocalization that convey the ‘non-serious nature’ of the interaction: ‘I want to play’ [8,40,91,92,93]. In canids, the play bow [94] (Figure 3) is a typical playful signal, mainly performed to reinitiate play after a pause [95] or to prolong a play session [80].

**Figure 4:**
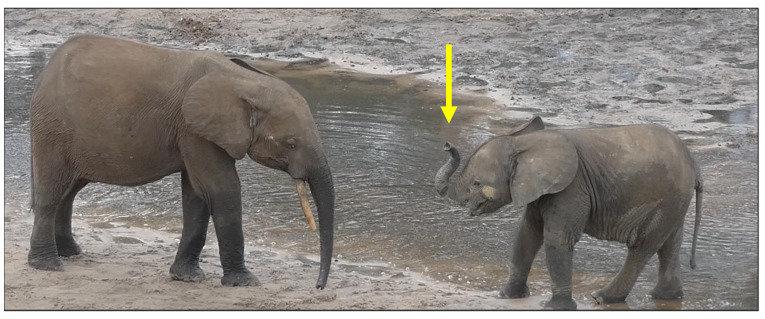
An example of the play trunk periscope (indicated by the yellow arrow), a play marker in elephant play. An elephant pauses and approaches a group mate with the trunk held up in a periscope or S-shape position [37,96]. The National Park of Dzanga-Sangha (Central African Republic). Edited by Giada Cordoni.

In elephants, kneeling on the front legs, waggling the head, and lifting and holding the trunk up in an S-shape are considered play markers [37,96] (Figure 4).

**Figure 5:**
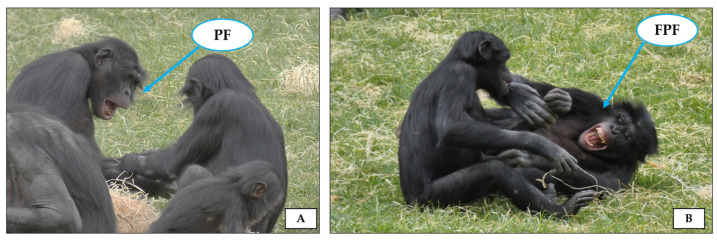
Pictures illustrating the two variants of the open mouth display (indicated by the blue arrows) performed during play in many non-human primates: (**A**) play face (PF), in which the mouth is opened and lower teeth are exposed, and (**B**) full play face (FPF), in which the mouth is opened and both lower and upper teeth are exposed. The group of bonobos (*Pan paniscus*) housed at La Vallée des Singes (Romagne, France). Photo by Giada Cordoni.

In juvenile rats, specific ultrasonic calls are emitted during playful sessions for different purposes: sustaining the individual’s playful mood, prolonging the interaction, and avoiding escalation of play into aggression [97]. In many mammals such as primates and carnivores, where the role of vision in communication is important (although not exclusive), the relaxed open mouth is the typical playful expression that has been largely used to investigate mood communication and exchange between players (for a review see [98]). In particular, in non-human primates the relaxed open mouth display has been described in two morphs: play face (PF; mouth opened and lower teeth exposed) and full play face (FPF; mouth opened and both lower and upper teeth exposed; [8,10] (Figure 5).

Some scholars consider PF and FPF homologous to the human smile and laughter, thus suggesting that these facial expressions evolved long before the appearance of modern humans [99]. Smile and laughter, and the non-human counterparts PF and FPF, are generally considered signals of a positive emotional state, although with different intensities [8,100,101,102,103,104]. But, for example, the human smile may not be solely indicative of a positive state (e.g., happiness), but, depending on context, it can communicate nervousness, need to please, embarrassment, or a welcoming attitude [105]. These different meanings can be associated with specific changes in the morphological and dynamic characteristics of the smile [106]. Human laugher cannot be considered exclusively a display of humour or happiness either [107,108]. Indeed, laughter can both regulate social relationships and limit social tension [107,108,109]. Symons [73] explains that ‘the only facial expression consistently observed in aggressive play is the relaxed open mouth face, or play-face’. Moreover, in non-human primates play faces have also been observed outside the play context. For example, qualitative data on long-tailed macaques (*M. fascicularis*) reported the presence of the play face during social interactions serving in lieu of direct aggression or as rank expression [110]. In bonobos, PF and FPF can be present during socio-sexual contacts, even if infrequently (mean proportion of playful expressions during socio-sexual contacts: 0.03 ± 0.02 SE [111]). Furthermore, the literature has mostly conflated PF and FPF into a single expression for analytical purposes, but in many species evolution has maintained the two morphs (i.e., PF and FPF [10]). Hence, from an adaptive standpoint, it is reasonable to hypothesize that these two signals may not serve entirely overlapping functions.

Based on the literature, FPF, compared to PF, may be more associated with high-intensity playful interactions to clearly communicate the non-aggressive intent of players, [112] and it may be more frequent in more tolerant species where the exposure of the upper teeth is less likely to be mistaken for a threat as in despotic species [9,113]. A recent paper demonstrated that in lowland gorillas PF and FPF differed from both a morphological and functional point of view [11]. While PF was followed by an increase in play session variability, FPF was associated with more asymmetric playful interactions. The use of a more evident signal (FPF) may better clarify the clear statement of purpose, thus permitting the playmates to switch play into a more competitive and cognitively demanding interaction, which, in turn, may enhance the self- and social-assessment process [75,114,115]. Similarly, in preschool children exaggerated laughter (more evident signals) can be most often linked with highly competitive forms of playful interactions [116].

Thus, from an evolutionary point of view, we may hypothesize that the use of more evident signals has been retained when it is necessary to elicit appropriate behavioural responses from partners and when the sharing of context (e.g., high-intensity play) may be crucial for minimizing the risk of misunderstanding. In the past decades, different freeware to investigate facial expressions (such as FACS and Openface for humans [117,118]) have been developed, which allows a fine analysis of the different expression morphs and facial units involved. Such tools allow future studies to extend the investigation of facial expressions possibly used during play beyond PF and FPF, and beyond their dichotomy, as they can be just two extremes of a graded expression [119]. Revisiting the old literature may be very helpful as in the past (where images and videos were not easy to produce) articles would delve deep into describing motor patterns and expressions. For example, in crab-eating macaques (*M. mulatta*) the exposure of the unpigmented eyelids is the mildest form of ‘pucker face’, an expression that can be predictive of non-agonistic approaches, including play [73]. Future investigation can increase the study of play signals in a nuanced way, to obtain more fine-tuned information on the emotional states that they may possibly convey. This process has started, and the time is now ripe to strengthen this research line.

## 6. Rapid Facial Mimicry: The Transfer of Mood, but What Mood?

**Figure 6:**
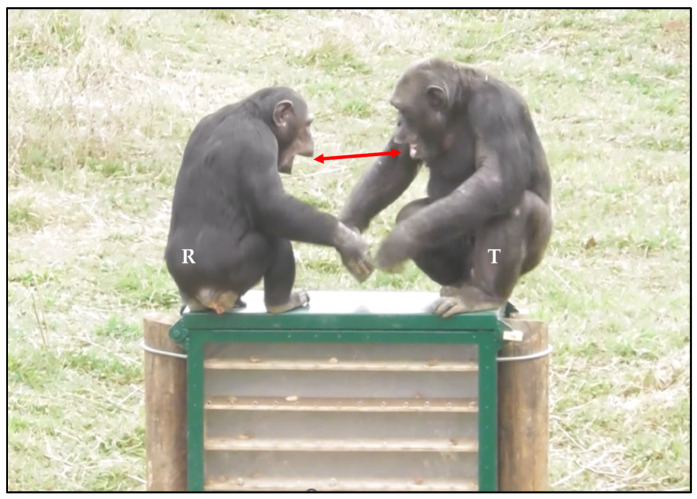
An example of rapid facial mimicry (RFM) between chimpanzees (*Pan troglodytes*) during a play fighting interaction. The exact same facial expression emitted by the trigger T (first stimulus) is replicated by the responder R within 1 s after the emission of the first stimulus (see the red arrow) [119]. Screenshot Giada Cordoni. Edited by Giada Cordoni.

During playful interactions, one player may rapidly (<1 s) and involuntarily replicate the facial expression performed by the partner (i.e., the trigger); this phenomenon is known as rapid facial mimicry (RFM; [120]; Figure 6).

From a neurological standpoint, RFM finds its roots in the automatic coupling of perception and action within the brain’s sensorimotor areas, as foreseen by the Perception–Action Model (PAM), possibly involving the mirror neuron system [121,122,123,124,125,126]. According to the PAM, observing another’s facial expression activates shared neural areas that enable replication not only of the expression but possibly also of the emotion it conveys. The coupling between motor replication and emotional replication may have occurred during evolution, starting from common external emotional stimuli eliciting similar reactions in the perceivers. This process may have then evolved so that the expression of one individual acts as a triggering stimulus for the replication of the same expression and underlying emotion by another individual [111]. In light of this, RFM is considered a possible manifestation of emotional contagion [124,127]. This topic is still under debate since, according to some scholars, facial expressions (i) are not always associated with internal states of individuals, (ii) can be generated in multiple emotional contexts, and (iii) are often not generated during extreme emotional experience [121]; furthermore, mimicry can be useful for reducing ambiguity, especially in species with competitive play fighting.

The question we raise here is how we can infer the possible emotional states that may be transferred via the rapid replication of facial expressions during play. If RFM is merely a motor replication phenomenon, one would expect it to be present in a comparable manner across all dyads within a social group. If RFM underlies something beyond the purely motor domain, it is possible that there are differences in the expression of RFM between different dyads. RFM, by promoting the sharing of an emotional state, could have repercussions on the interaction [109]. Indeed, RFM may be modulated by contextual and social factors (e.g., group membership and cooperative priming [128]) and can be linked, particularly in non-human primates, to longer and more intense playful sessions [27,129,130,131,132,133].

Originally, RFM was associated with individual positive emotional states favoring inter-individual cohesion within the group [8,134,135]. For example, in domestic dogs RFM is more frequent in response to friends, then acquaintances, and lastly strangers [8]. In geladas (*Theropithecus gelada*), RFM occurs significantly more between mother–infant pairs [134]. Nevertheless, it is worth noting that negative expressions of emotional states can also be rapidly replicated; RFM may not always lead to an increase in social cohesion and the mimicker can gain benefits for themselves primarily [136].

According to the ‘decreasing predictor error’ hypothesis, via mimicry an individual may be more like the partner and thus can predict the partner more easily. This leads to benefits primarily for the mimicker and, as an outcome of mimicry, for social interaction strength [136]. To our knowledge, up to the present, there is no evidence in primates of an increase of RFM following the gradient of social bond strength (acquaintances, friends, and close kin), known as the empathy gradient [122]. On the contrary, a study found that in young gorillas the closeness of social bonds negatively influenced the occurrence of RFM [132]. Similarly, in young toddlers, another study found an inverse relationship between affiliation levels and frequency of RFM [137]. In three species of spider monkey (*Ateles fusciceps*, *A. hybridus*, and *A. paniscus*), RFM was not modulated by both individual and social factors, possibly because of the fluid social dynamics that characterize spider monkeys or because RFM may help motoric synchronization per se [27].

While RFM can be associated with longer play sessions, as explained above, it may also be associated with a wider array of different types of more intense offensive playful patterns (such as biting, pushing, slapping, and pulling) compared to the single, unreplicated play face [27]. Also, in African elephants, the motor replication of trunk movements signalling play was associated with more offensive play patterns [138]. In this respect, automatic motor mimicry may help reduce the risk of misinterpreting behavioural patterns while simultaneously promoting a more competitive aspect of playful interactions, all within the context of ensuring safety.

Based on these pieces of evidence, automatic motor mimicry in general may be linked to different emotional states and have various functions, from building to maintaining social interactions, or even preserving them under challenging and otherwise competitive relations. Hence, the study of motor mimicry can represent a valid method for evaluating the emotional states of individuals engaging in play. The need for motor and/or emotional synchronization with other individuals, or with certain individuals rather than others, may vary depending on the context. It is reasonable to assume that automatic motor mimicry has evolved to be activated in a flexible and functional manner in response to socio-ecological contexts that change over time.

## 7. Conclusions

In conclusion, this review selects and combines the studies on play behaviour in an innovative way, presenting the methods (e.g., play indices and social network analysis), tools (e.g., sequential analysis and facial coding software), and evidence that indicate the emotional state underlying play, which is much more complex than previously thought. Play is a nuanced behaviour shifting between cooperation and competition and between positive and negative emotional states. These emotional states can be better understood by quantitatively determining the structural and communicative features of play as well as its neuro-hormonal and physiological correlates. It is crucial to consider the context and dynamics of play (from the socio-ecological setting to the exchange of individual motor patterns and signals) to understand the emotional states underlying it. This approach is only in its infancy, and much remains to be done. We believe this review can lay the groundwork for future studies aimed at developing scientific methods to detect, from a behavioural perspective, the affective nuances behind playful interactions.

## Data Availability

Not applicable because no new data were created.
